# The organic ester *O,O’*-diethyl-(*S,S*)-ethylenediamine-*N,N’*-di-2-(3-cyclohexyl)propanoate dihydrochloride attenuates murine breast cancer growth and metastasis

**DOI:** 10.18632/oncotarget.25610

**Published:** 2018-06-15

**Authors:** Milena Jurisevic, Aleksandar Arsenijevic, Jelena Pantic, Nevena Gajovic, Jelena Milovanovic, Marija Milovanovic, Jelena Poljarevic, Tibor Sabo, Danilo Vojvodic, Gordana D. Radosavljevic, Nebojsa Arsenijevic

**Affiliations:** ^1^ Center for Molecular Medicine and Stem Cell Research, Faculty of Medical Sciences, University of Kragujevac, Kragujevac, Serbia; ^2^ Department of Pharmacy, Faculty of Medical Sciences, University of Kragujevac, Kragujevac, Serbia; ^3^ Department of Histology and Embryology, Faculty of Medical Sciences, University of Kragujevac, Kragujevac, Serbia; ^4^ Faculty of Chemistry, University of Belgrade, Belgrade, Serbia; ^5^ Institute of Medical Research, Faculty of Medicine, Military Medical Academy, Belgrade, Serbia

**Keywords:** *O,O’*-diethyl-(*S,S*)-ethylenediamine-*N,N’*-di-2-(3-cyclohexyl)propanoate dihydrochloride, breast cancer growth, metastasis, apoptosis, proliferation

## Abstract

Pharmacological treatment of cancer is mostly limited by drug-toxicity and resistance. It has been noticed that new organic ester ligand, *O,O’-*diethyl-(*S,S*)-ethylenediamine-*N,N’*-di-2-(3-cyclohexyl)propanoate dihydrochloride (named DE-EDCP) showed effective cytotoxic capacities against several human and mouse cancer cell lines. However, its effects on tumor growth and metastasis are unexplored. The aim of present study was to examine the ability of DE-EDCP to inhibit 4T1 murine breast cancer growth and progression and to explore possible molecular mechanisms. DE-EDCP exhibited significant tumoricidal activity on human and murine breast cancer cell lines. Further, marked reduction of murine breast cancer growth and progression by DE-EDCP was shown. DE-EDCP exhibits fewer side-effects compared to cisplatin as a conventional chemotherapeutic. Results obtained from *in vivo* and *in vitro* experiments indicate that DE-EDCP induces apoptosis and inhibits proliferation of 4T1 cells. DE-EDCP increases percentage of 4T1 cells in late apoptosis, expression of pro-apoptotic Bax and caspase-3, while decreases expression of anti-apoptotic Bcl-2. DE-EDCP treatment increased the percentage of TUNEL-positive nuclei and reduced Ki-67 expression in breast cancer tissue. DE-EDCP decreased expression of cyclin D3 and Ki-67, increased expression of cyclin-dependent kinase inhibitors p16, p21 and p27 and arrested 4T1 cells in G0/G1 cell cycle phase. Expression of STAT3 and downstream regulated molecules, NANOG and SOX2, was reduced in 4T1 cells after DE-EDCP treatment.

In conclusion, DE-EDCP impairs breast cancer growth and progression by triggering cancer cell death and inhibition of cancer cell proliferation. DE-EDCP might be of interest in the development of the new anticancer agent.

## INTRODUCTION

Cancer is on its way to become number one killer across the world [1]. Platinum-based drugs are one of the mostly used anticancer agents [2, 3]. Cisplatin or cis-diamminedichloroplatinum(II) is the most widely known metal-based anticancer drug, used for treatment of a variety of malignancies, including breast cancer [4]. However, clinical utility of cisplatin has been often limited due to toxicity [5–7] and acquired or intrinsic resistance [8–10]. The lack of efficiency caused by these limiting factors is the main reason why vigorous attempts are committed to develop novel platinum complexes which might overcome the shortcomings of cisplatin.

Large number of edda (ethylenediamine-*N,N’*-diacetate)-type ligands and their corresponding platinum complexes have been successfully synthesized [11, 12] and some of them showed significant cytotoxic effects [13].

It has been shown that platinum(IV) [14] and platinum(II) [15] complexes with cyclohexyl-functionailized ehylenediamine*-N,N’*-diacetate-type ligand exhibit effective tumoricidal capacities against various cancer cell lines. The cytotoxic effects of these platinum complexes may be at least partly related to their organic ligands (ester derivatives of (*S,S*)-ethylenediamine-*N,N′-*di-2-(3-cyclohexyl)propanoic acid). Moreover, the organic ligands alone demonstrated significant toxicity *in vitro*, towards a panel of different mice and human cancer cell lines [14–16]. *O,O’*-diethyl-(*S,S*)-ethylenediamine-*N,N’*-di-2-(3-cyclohexyl)propanoate dihydrochloride was synthesized as one of novel ester derivates of (*S,S*)-ethylenediamine-*N,N′-*di-2-(3-cyclohexyl)propanoic acid [14–16]. Among different organic ligands, *O,O’*-diethyl-(*S,S*)-ethylenediamine-*N,N’*-di-2-(3-cyclohexyl)propanoate dihydrochloride exhibited considerably similar or higher cytotoxic activity than cisplatin [14–16].

However, possible *in vivo* anticancer effects of new synthesized organic ester *O,O’*-diethyl-(*S,S*)-ethylenediamine-*N,N’*-di-2-(3-cyclohexyl)propanoate dihydrochloride (compound marked as DE-EDCP), has not been reported. The aim of our study was to investigate the effect of DE-EDCP on 4T1 murine breast cancer growth and progression, as well as possible molecular mechanism(s) of action.

## RESULTS

### DE-EDCP exerts cytotoxic capacity against mammary carcinoma cells

**Figure 1:**
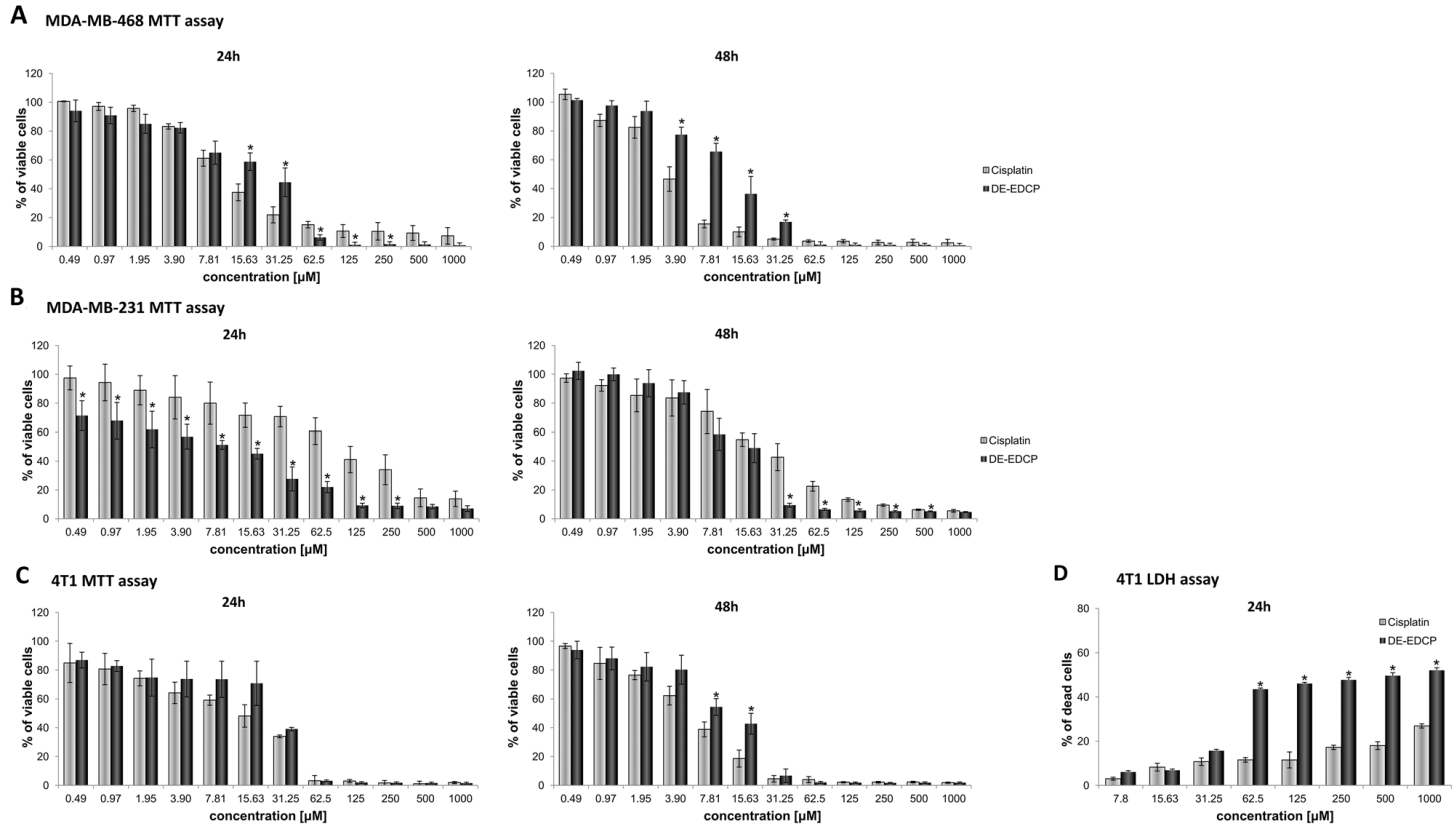
Dose-dependent cytotoxicity of DE-EDCP against human and murine breast cancer cells **(A, B)** Effect of DE-EDCP on viability of MDA-MB-468 and MDA-MB-231 cells after 24h and 48h analyzed with the MTT assay. **(C)** The viability of 4T1 cells treated with DE-EDCP and cisplatin for 24h and 48h was evaluated using MTT assay. **(D)** LDH assay of 4T1 cells treated with DE-EDCP and cisplatin for 24h. All data are presented as mean values ± SD from three independent experiments performed in triplicates. ^*^ p < 0.05 DE-EDCP vs. cisplatin treated cells.

The effects of DE-EDCP on viability of several breast cancer cells (murine 4T1 and human MDA-MB-231 and MDA-MB-468) were examined using MTT assay. Cell viability was tested after treatment with growing concentrations of DE-EDCP for 24 and 48 hours, and IC_50_ values were calculated. The obtained data showed that DE-EDCP decreased viability of all tested tumor cell lines in a dose-dependent manner (Figure 1).

Cytotoxic potential of DE-EDCP toward MDA-MB-468 cells was significantly lower compared to cisplatin in concentrations 15.63-31.25 μM after 24 hours of exposure and in concentrations 3.90-31.25 μM after 48 hours treatment (Figure 1A). Cytotoxicity of DE-EDCP toward MDA-MB-231 was higher after 24 hours treatment in comparison with 48 hours exposure (Figure 1B). Further, viability of MDA-MB-231 cells was significantly reduced after 24 hours treatment with DE-EDCP in comparison with the same treatment with cisplatin (Figure 1B). Cytotoxicity of DE-EDCP against MDA-MB-231 cells after 48 hours exposure was higher than activity of cisplatin for concentrations 31.25-500 μM (Figure 1B).

As shown in Figure 1C, at concentrations of 31.25 μM or higher, DE-EDCP exerted the effective dose-dependent cytotoxic effect against 4T1 cells. The treatment of 4T1 cells with tested substances for 24 hours resulted in approximately equal cytotoxic capacity of DE-EDCP and referent cytostatic cisplatin in concentrations 62.5-1000 μM, and more importantly in the lowest concentrations 0.49-3.90 μM (Figure 1C). The cytotoxicity of tested compound after 48 hours treatment was lower in comparison with the effect of cisplatin in concentration range from 7.81 to 15.63 μM (Figure 1C).

The IC_50_ values of DE-EDCP against 4T1 and MDA-MB-468 are similar to those for cisplatin, while IC_50_ of DE-EDCP against MDA-MB-231 is several times lower following 24 hours treatment (Table 1).

**Table 1:** The IC_50_ values of DE-EDCP and cisplatin determined by MTT assay

Compound	IC_50_ ± SD (μM)					
	MDA-MB-468		MDA-MB-231		4T1	
	*24 hours*	*48 hours*	*24 hours*	*48 hours*	*24 hours*	*48 hours*
Cisplatin	16.68±1.83	5.01±1.03	66.01±0.69	20.71±0.32	10.59±0.36	7.97±0.56
DE-EDCP	16.52±2.78	15.58±2.94	5.14±0.63	17.45±3.33	12.90±0.75	12.03±2.86

Data are presented as mean values ± SD from three experiments.

The LDH assay confirmed the significant cytotoxic effect of DE-EDCP against 4T1 cells (Figure 1D). The results revealed that the level of LDH release was increased after exposure of 4T1 cells to DE-EDCP for 24 hours compared to cells treated with cisplatin, indicating that DE-EDCP could affect the cell membrane integrity. Additionally, DE-EDCP increased the release of LDH in a dose-dependent manner. The LDH levels increased from 15.67% to 52.02% following DE-EDCP treatment in comparison with 10.73% to 26.89% after cisplatin treatment at concentration range from 62.5 to 1000 μM.

### DE-EDCP reduces tumor growth and metastasis

In view of the pronounced tumoricidal effects of DE-EDCP *in vitro*, the next goal of present study was to examine the ability of tested compound to inhibit murine breast cancer growth and progression *in vivo*. 4T1 cells were orthotopically implanted into the mammary fat pad of mice. After the appearance of palpable tumor, mice were treated with DE-EDCP, cisplatin or vehicle.

**Figure 2:**
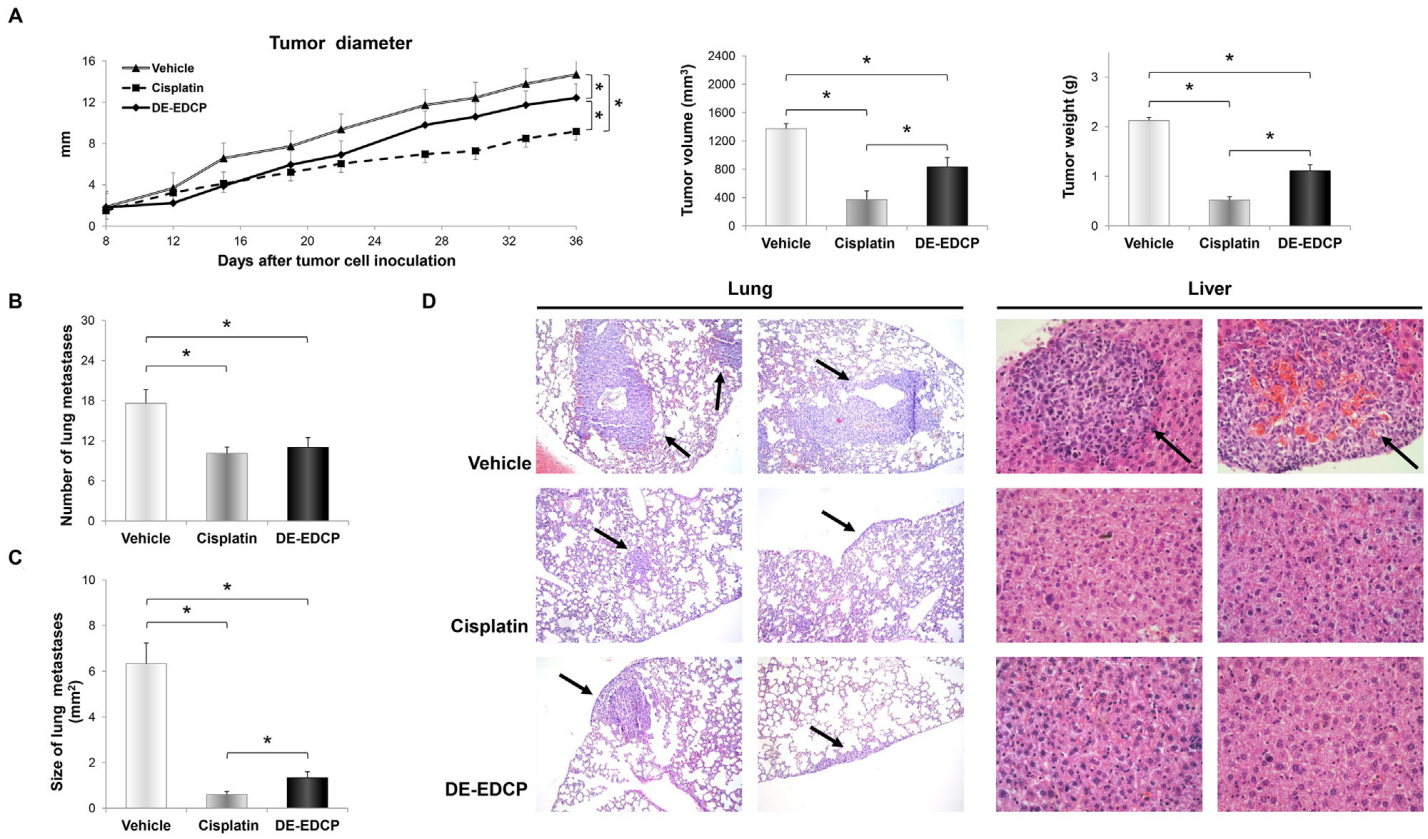
DE-EDCP treatment of mice bearing breast cancer inhibits tumor growth and progression **(A)** Tumor diameter, volume and weight in mice treated with DE-EDCP, cisplatin or phosphate-buffered saline (vehicle) (n=7-8 animals per group) (36^th^ day of experiment:^*^ tumor diameter-: DE-EDCP vs. vehicle p=0.005; DE-EDCP vs. cisplatin p=0.001; cisplatin vs. vehicle p=0.000; tumor volume-: DE-EDCP vs. vehicle p=0.03; DE-EDCP vs. cisplatin p=0.005; cisplatin vs. vehicle p=0.002; tumor weight-: DE-EDCP vs. vehicle p=0.004; DE-EDCP vs. cisplatin p=0.001; cisplatin vs. vehicle p=0.002). **(B, C)** Number and size of lung metastasis from treated mice. Data are presented as mean ± SE (^*^ number of lung metastasis-: DE-EDCP vs. vehicle p=0.032; cisplatin vs. vehicle p=0.008; size of lung metastasis-: DE-EDCP vs. vehicle p=0.009; DE-EDCP vs. cisplatin p=0.029; cisplatin vs. vehicle p=0.002). **(D)** Representative hematoxylin and eosin staining of lung and liver tissue sections showing metastatic colonies (arrows) (magnification at x100 and x400).

The short-course treatment with DE-EDCP (10 mg/kg body weight/5 doses per cycle/2 cycles during 12 days), started from the day 5 after tumor cell implantation, was associated with the significant reduction of breast cancer growth compared with vehicle treated animals (Figure 2A). Until day 22, DE-EDCP exhibited similar effects on tumor growth as cisplatin. After that point, although application of cisplatin showed better effects, tumor growth in DE-EDCP treated mice was significantly slower to the end of experiment (day 36 following inoculation of tumor cells) compared to vehicle treated animals (Figure 2A). Furthermore, tumor volume and weight, measured after necropsy, were markedly lower in mice treated with DE-EDCP in comparison to vehicle treated mice (Figure 2A).

On day 36, the pulmonary metastases were detected in all injected animals, but the number of metastatic colonies was significantly decreased by 1.6-fold in DE-EDCP treated and 1.7-fold in cisplatin treated mice when compared to control animals (Figure 2B). Furthermore, DE-EDCP as well as cisplatin effectively reduced the size of metastases (4.8-fold and 10.55-fold, respectively) (Figure 2C and 2D). Metastatic colonies were not detected in the liver of mice treated with DE-EDCP or cisplatin, whereas three out of seven vehicle treated mice (42.1%) developed liver metastases (Figure 2D).

### Tumor cells treated with DE-EDCP undergo apoptotic cell death

This experimental study indicates that DE-EDCP exhibits beneficial anticancer capacity in 4T1 murine breast cancer model. Therefore, the following aim was to identify and characterize the mechanisms implicated in DE-EDCP- induced inhibition of breast cancer progression.

**Figure 3:**
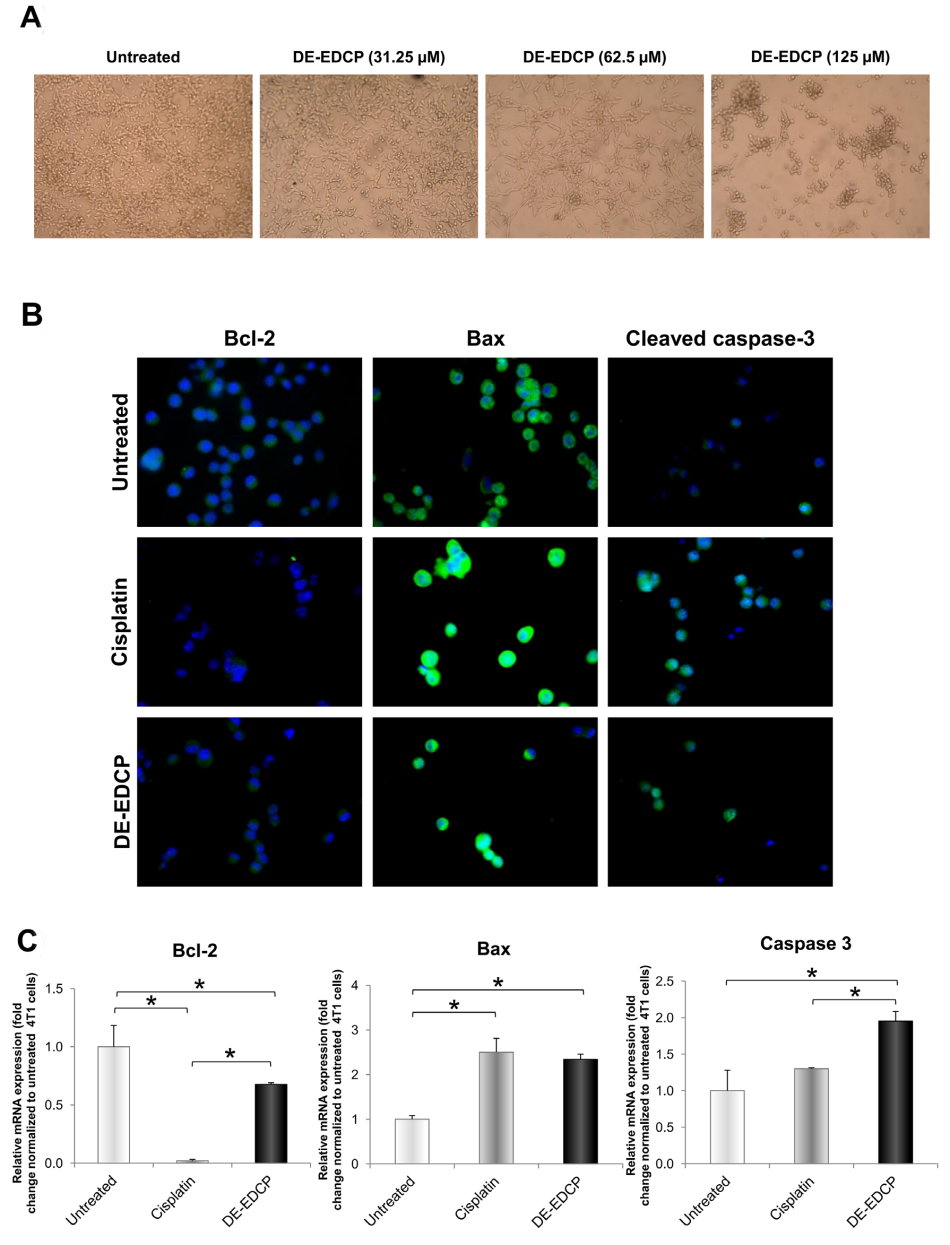
Morphological changes and expression of key apoptosis-related molecules in 4T1 cells after DE-EDCP treatment **(A)** Morphological changes of 4T1 cells exposed to various concentrations of DE-EDCP for 24h. **(B)** Immunofluorescence staining for Bcl-2 (green), Bax (green) and cleaved caspase-3 (green) together with DNA staining with DAPI (blue) in 4T1 cells incubated with DE-EDCP or cisplatin (31.25 μM) for 24h, as well as in untreated cells (magnification at x200). **(C)** mRNA expression of Bcl-2, Bax and caspase-3 quantified by RT-PCR in 4T1 cells after DE-EDCP 24h treatment. DE-EDCP treatment markedly increased the expression of Bax and caspase-3 mRNA and decreased the expression of Bcl-2 mRNA in 4T1 cells. β-actin mRNA was used as an internal control. Data points are represented by the expression ratio and mean±SD fold of control in 4T1 cells. (^*^ Bcl-2-: DE-EDCP vs. untreated p=0.03; DE-EDCP vs. cisplatin p=0.006; cislatin vs. untreated p=0.001; Bax-: DE-EDCP vs. untreated p=0.011; cislatin vs. untreated p=0.009; caspase-3-: DE-EDCP vs. untreated p=0.015; DE-EDCP vs. cisplatin p=0.021)

Twenty four hours after the treatment with DE-EDCP at growing concentrations, it was evident that 4T1 cells became rounded and detached (Figure 3A), suggesting that the cancer cells underwent apoptosis. Likewise, the treatment with cisplatin induced similar cell changes (data not shown). Expression of key apoptosis-related molecules, Bcl-2, Bax and cleaved caspase-3 in 4T1 cells following treatment with DE-EDCP or cisplatin was visualized by immunofluorescence staining. Moderately lower expression of anti-apoptotic Bcl-2 was observed in cells treated with DE-EDCP compared to untreated cells (Figure 3B), while cisplatin clearly downregulates the expression of Bcl-2. In contrast, expression of both pro-apoptotic Bax and cleaved caspase-3 was increased after DE-EDCP or cisplatin treatment, compared to untreated 4T1 cells. Quantitative analysis of Bcl-2, Bax and caspase-3 mRNA levels is in accordance with immunofluorescence staining analysis. Bax mRNA levels were significantly increased in DE-EDCP and cisplatin treated compared to untreated 4T1 cells (Figure 3C). In addition, DE-EDCP, but not cisplatin, increased the expression of caspase-3 mRNA level compared to untreated cells (Figure 3C). The caspase-3 mRNA level was significantly higher in DE-EDCP compared to cisplatin treated cells (Figure 3C). Bcl-2 mRNA level was significantly reduced after DE-EDCP and cisplatin treatment (Figure 3C). The lowest Bcl-2 mRNA level was observed in cisplatin treated cells.

**Figure 4:**
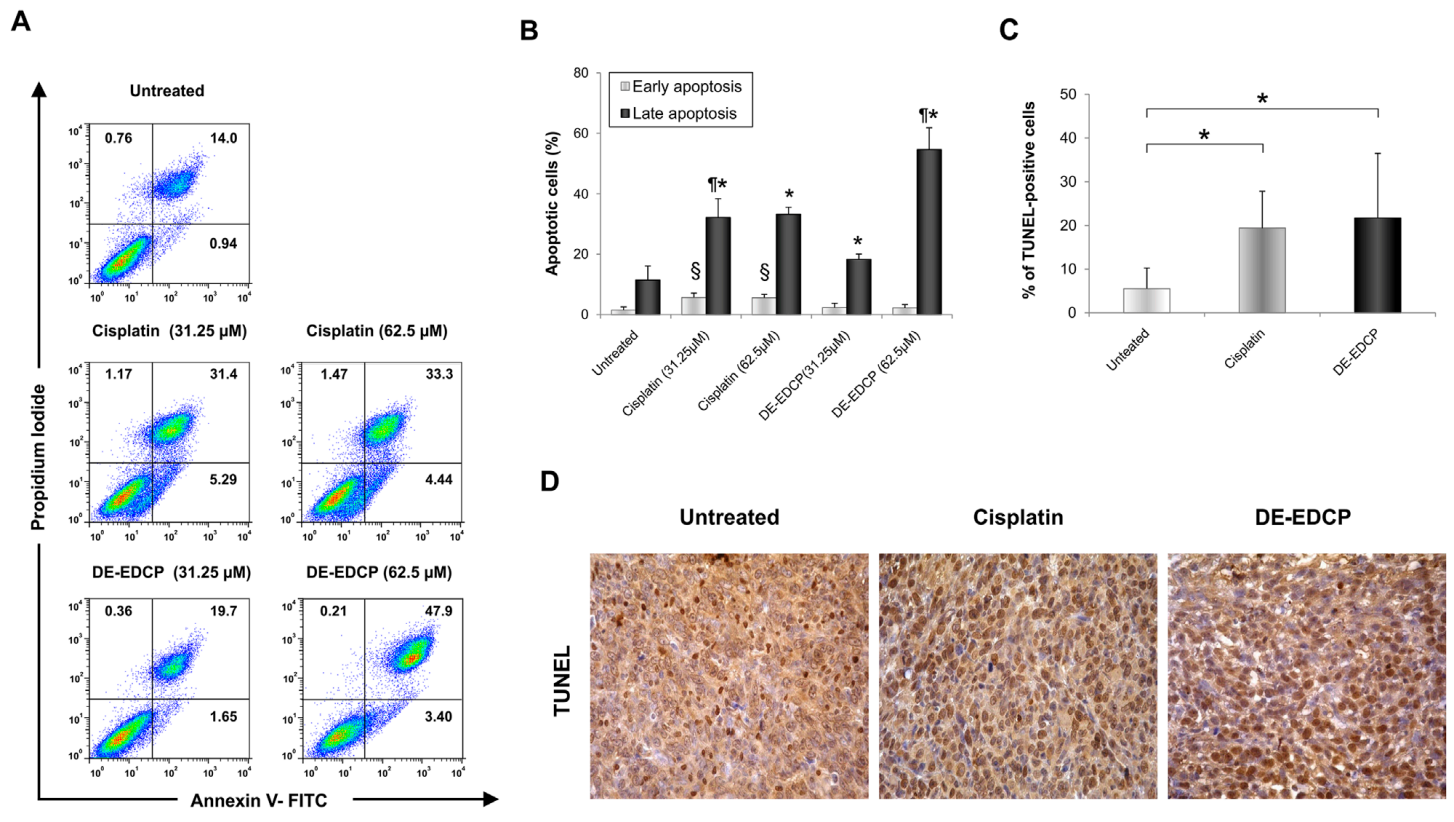
DE-EDCP enhances tumor cell apoptosis in murine breast cancer **(A)** Representative dot plots illustrate population of viable (AnnV^-^ PI^-^), early apoptotic (Ann V^+^ PI^-^), late apoptotic (AnnV^+^ PI^+^) and necrotic (AnnV^-^ PI^+^) cells. **(B)** Apoptosis of untreated as well as DE-EDCP and cisplatin (31.25 and 62.5 μM) treated 4T1 cells were analyzed by flow cytometry using Annexin V (FITC) and PI double staining. The data are presented as means ± SD of a three independent experiment, *p<0.05* significantly different from: ^*^untreated vs. DE-EDCP or cisplatin treated cells; ¶ DE-EDCP vs. cisplatin treated cells; § cisplatin vs. untreated or DE-EDCP treated cells. **(C)** Quantitative analysis of the rate of apoptosis: TUNEL-positive nuclei (brown) were counted in five random fields, and the data were summarized as the mean percentage of positive cells. Data are presented as mean ± SD from four tumors per group, (^*^ DE-EDCP vs. untreated p=0.028; cisplatin vs. untreated p=0.048). **(D)** TUNEL assay in breast cancer tissues at 36^th^ day (magnification at x400).

To quantify the apoptotic death of 4T1 cells induced by DE-EDCP, Annexin V (AnnV) /Propidium iodide (PI) double staining was performed. Growing cell cultures were exposed to DE-EDCP or cisplatin at concentrations of 31.25 and 62.5 μM for 24 hours. As illustrated in Figure 4A and 4B, the majority of apoptotic cells were phenotyped as AnnV^+^/PI^+^ indicating late apoptosis. The obtained data showed that both DE-EDCP and cisplatin induced significantly increased late apoptotic cell death in comparison to untreated cells, while the highest percentage of early apoptotic cells was noticed following cisplatin treatment (Figure 4B). Further, at lower concentration (31.25 μM), cisplatin induced late apoptosis more efficiently than DE-EDCP. However, the highest percentage of late apoptotic cells was observed after DE-EDCP treatment at the concentration of 62.5 μM (Figure 4B). The percentage of late apoptotic cells treated with 62.5 μM of DE-EDCP was markedly increased (54.63% ±7.19) in comparison to cisplatin treated cells (33.23±2.30) (Figure 4B).

In order to test consistency of proapoptotic effect of DE-EDCP *ex vivo*, we used TUNEL (terminal deoxynucleotidyl transferase-mediated dUTP labeling) assay for *in situ* detection of apoptosis-triggered DNA fragmentation in tumor tissue. As shown in Figure 4C and 4D, DE-EDCP and cisplatin treated tumors have more TUNEL-positive cells than vehicle treated tumors.

### DE-EDCP inhibits proliferation of breast cancer cells

**Figure 5:**
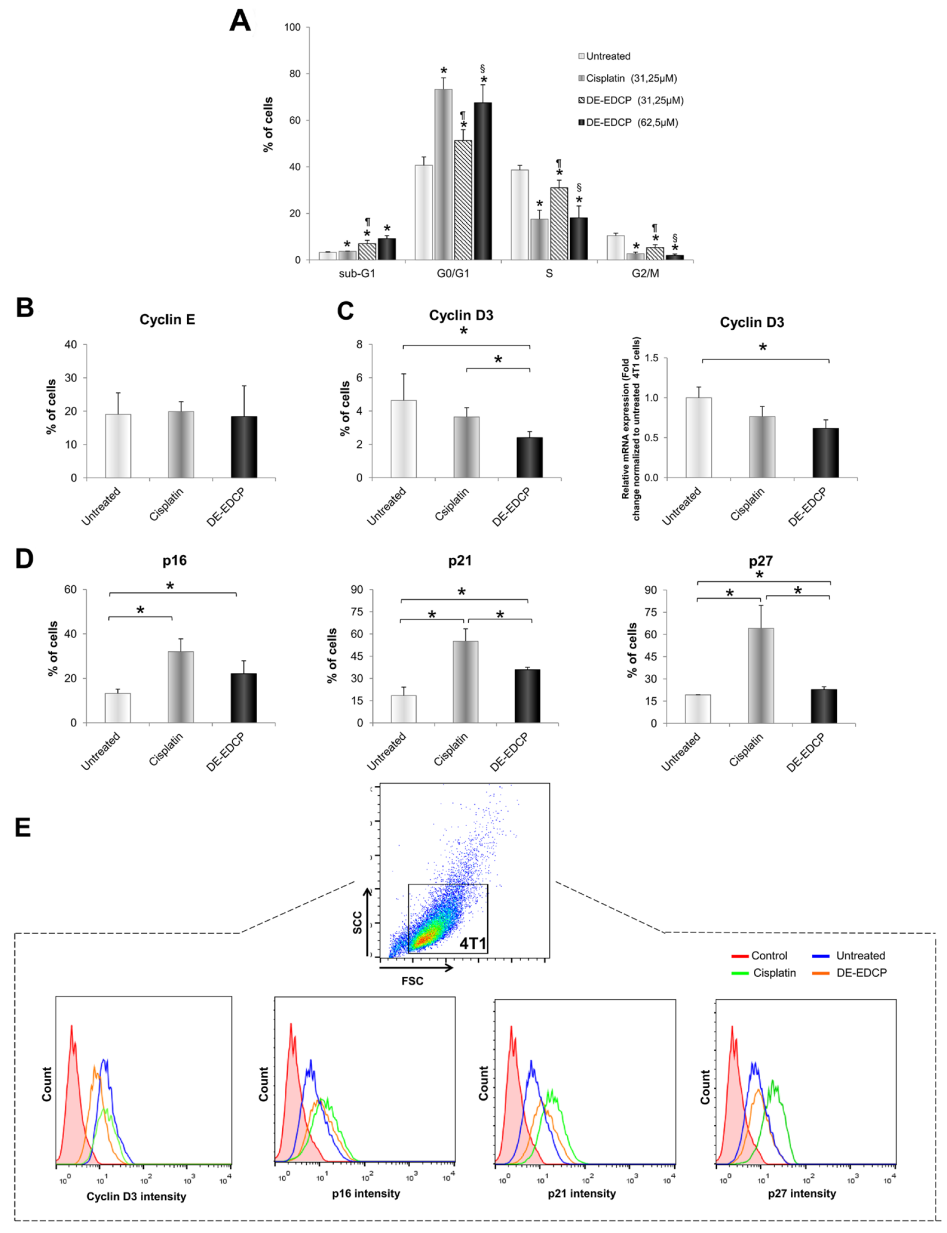
DE-EDCP induces cell cycle arrest at the G0/G1 checkpoint in 4T1 cells **(A)** 4T1 cells cycle analyzed by flow cytometry. Results are expressed as the percentage of cells in different phases of the cell cycle. Data are presented as the mean ± SD, *p*<0.05 significantly different from: ^*^untreated vs. DE-EDCP or cisplatin treated cells; ¶ DE-EDCP vs. cisplatin treated cells; § DE-EDCP treated cells lower and upper concentration. **(B)** Analysis of cyclin E expression in 4T1 cells exposed to DE-EDCP or cisplatin (31.25 μM) for 24h using flow cytometry by first gaiting out cell debris and cell clumps in forward/side scatter plot. Data are presented as mean ± SD. **(C)** Analysis of cyclin D3 expression in 4T1 cells exposed to DE-EDCP or cisplatin (31.25 μM) for 24h using flow cytometry by first gaiting out cell debris and cell clumps in forward/side scatter plot. Data are presented as mean ± SD (^*^ DE-EDCP vs. untreated p=0.002; DE-EDCP vs. cisplatin p=0.01). mRNA expression of cyclin D3 quantified by RT-PCR in 4T1 cells after DE-EDCP (31.25 μM) treatment. β-actin mRNA was used as an internal control. Data points are represented by the expression ratio and mean±SD fold of control in 4T1 cells (^*^ DE-EDCP vs. untreated p=0.017). **(D)** Analysis of p16, p21 and p27 expression in 4T1 cells exposed to DE-EDCP or cisplatin (31.25 μM) for 24h using flow cytometry by first gaiting out cell debris and cell clumps in forward/side scatter plot. Data are presented as the mean ± SD (^*^ p16-: DE-EDCP vs. untreated p=0.041; cisplatin vs. untreated p=0.034; p21-: DE-EDCP vs. untreated p=0.002; DE-EDCP vs. cisplatin p=0.017; cisplatin vs. untreated p=0.001; p27-: DE-EDCP vs. untreated p=0.031; DE-EDCP vs. cisplatin p=0.006; cisplatin vs. untreated p=0.004). **(E)** Representative histograms after flow cytometric analysis of cyclin D3, p16, p21 and p27 of three independent experiments are shown.

We next investigated whether, beside apoptosis, DE-EDCP inhibits cancer cell proliferation. Consequently, the cell cycle profile of 4T1 cells was determined after exposure to DE-EDCP or cisplatin for 12 hours. DE-EDCP (31.25 μM and 62.5 μM) or cisplatin (31.25 μM) treatment significantly increased the percentage of cells in G0/G1 phase in comparison with untreated cells (Figure 5A). Furthermore, the percentage of cells in S and G2/M phases was decreased after DE-EDCP and cisplatin treatment (Figure 5A). In addition, the significant increase in the percentage of cells in sub-G1 phase was found after the exposure to DE-EDCP (31.25 μM and 62.5 μM) (Figure 5A). Overall, the obtained data indicated that DE-EDCP inhibited cell proliferation through arrest of cell cycle progression in the G0/G1 phase and subsequent induction of apoptosis in 4T1 cells. DE-EDCP was more effective at higher concentration (62.5 μM), while cisplatin achieved similar effect at a concentration as low as 31.25 μM.

Further, we examined the expression of cell-cycle progression regulators by flow cytometry. DE-EDCP (31.25 μM) or cisplatin (31.25 μM) treatment did not affect the expression of cyclin E (Figure 5B). However, DE-EDCP treatment significantly reduced cyclin D3 expression (2.4%±0.37) compared to cisplatin treated (3.64%±0.56) and untreated cells (4.64%±1.59) (Figure 5C and 5E). In line with this finding, marked decrease of cyclin D3 mRNA level was observed after DE-EDCP treatment compared to untreated cells (Figure 5C).

Both DE-EDCP and cisplatin, enhanced the expression of inhibitor of cyclin D-CDK4 complex, p16, compared to untreated 4T1 cells (Figure 5D and 5E), thus contributing to cell cycle arrest at G0/G1 phase. Also, DE-EDCP and cisplatin markedly upregulated the expression of kinase inhibitor protein (KIP) family members, p21 and p27, compared to untreated 4T1 cells (Figure 5D and 5E). The expression of p21 and p27 was increased in cisplatin compared to DE-EDCP treated cells. These results suggested that the downregulation of cyclin D3 and the upregulation of p16, p21 and p27 by DE-EDCP led to cell cycle arrest at G0/G1 phase, and consequently resulted in growth cell inhibition.

### DE-EDCP decreases expression of Ki-67 and STAT3

**Figure 6:**
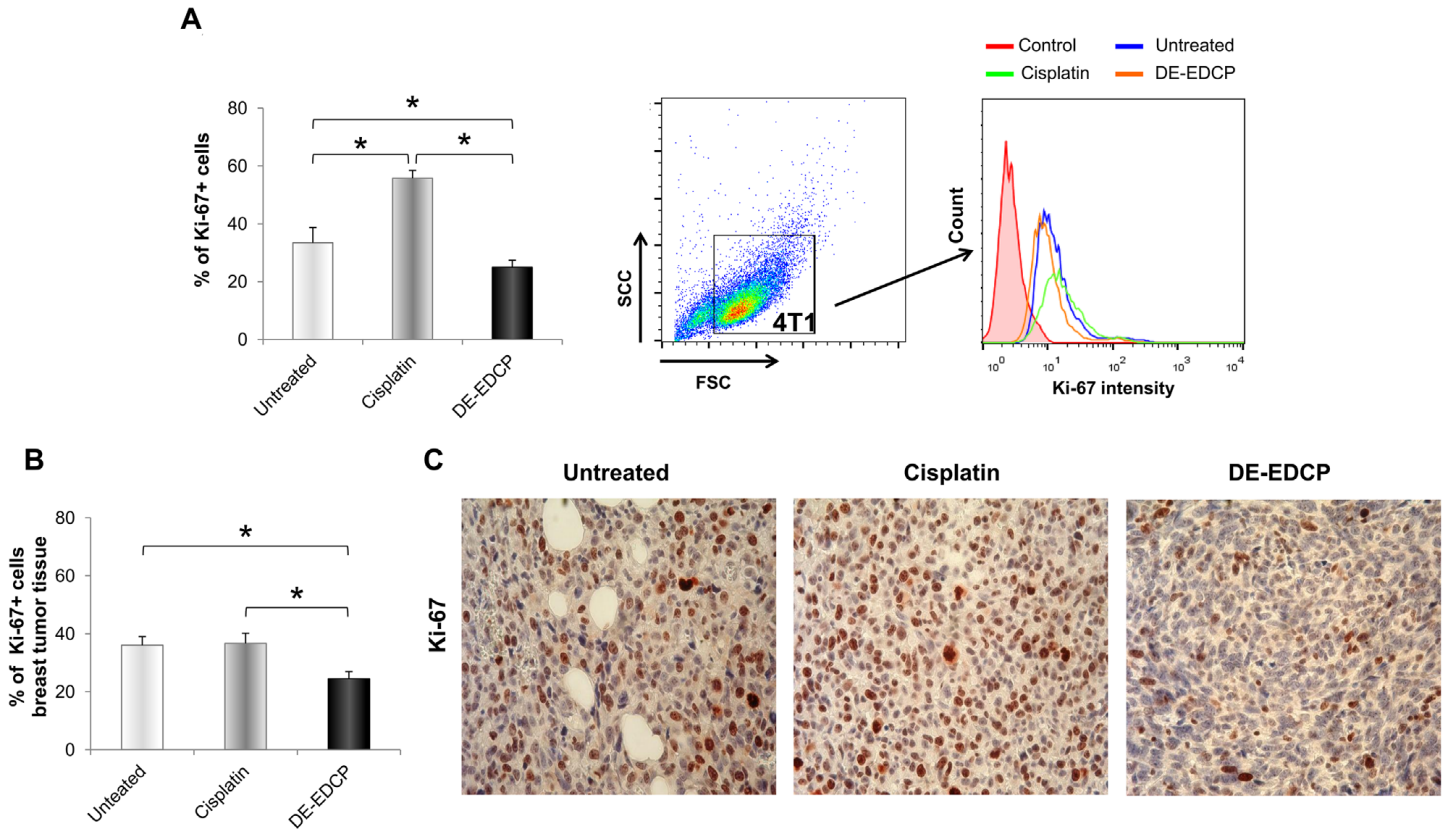
DE-EDCP treatment attenuates expression of Ki-67 in murine breast cancer **(A)** Analysis of Ki-67 expression in 4T1 cells exposed to DE-EDCP or cisplatin (31.25 μM) for 24h using flow cytometry by first gaiting out cell debris and cell clumps in forward/side scatter plot. Data are presented as the mean ± SD, (^*^ DE-EDCP vs. untreated p=0.020; DE-EDCP vs. cisplatin p=0.002; cisplatin vs. untreated p=0.009). Representative histograms of three independent experiments are shown. **(B, C)** At 36^th^ day of the experiment, tumors were harvested from tumor-bearing mice treated with DE-EDCP, cisplatin and vehicle and Ki-67 expression was detected using immunohistochemical method. Representative images and quantitative analysis of the percentage of Ki-67- positive cells are shown. Ki-67-positive cells were counted in five random fields (magnification at x 400), and data were summarized as the mean percentage of positive cells (four tumors per group). Data are presented as mean ± SE. (^*^DE-EDCP vs. untreated p=0.006; DE-EDCP vs. cisplatin p=0.004)

A recent study revealed that Ki-67 and STAT3 might be promising therapeutic targets in cancer, including breast cancer [17, 18]. As evaluated by flow cytometry, percentage of Ki-67-positive 4T1 cells treated with DE-EDCP (31.25 μM) was significantly decreased (24.97%±2.42) compared to untreated (33.43%±5.28) and cisplatin treated cells (55.77%±2.70) (Figure 6A). Also, there was significant increase of Ki-67 positive cells treated with cisplatin compared with untreated cells (Figure 6A). Further, immunohistochemistry showed decreased percentage of Ki-67-positive cells (24.46%±2.45) within breast cancer tissue of DE-EDCP treated mice compared to vehicle and cisplatin treated animals (36.05%±2.94 and 36.66%±3.47, respectively) (Figure 6B and 6C). There was no difference in the expression of Ki-67 in breast cancer tissue from mice treated with cisplatin or vehicle controls.

**Figure 7:**
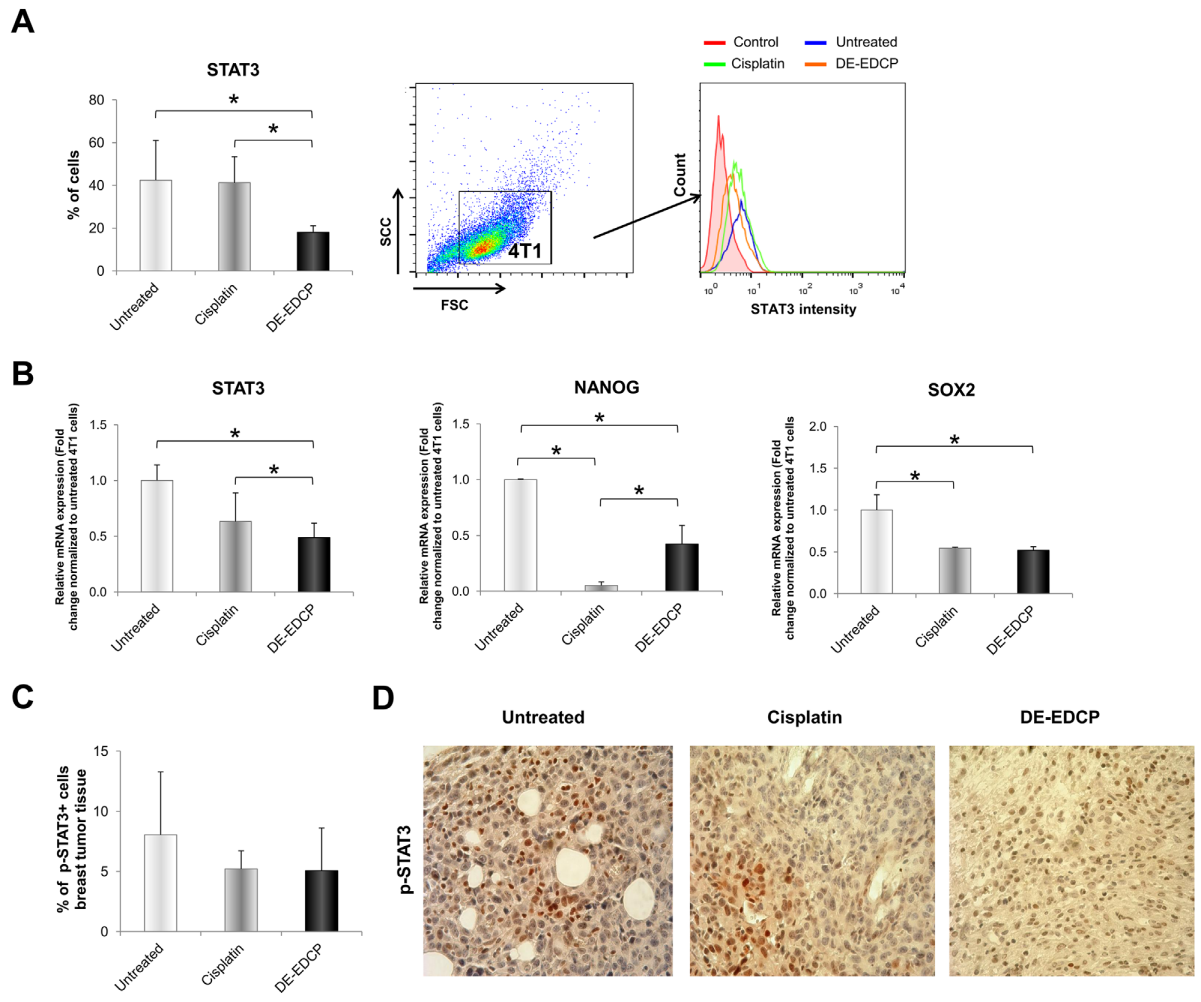
DE-EDCP treatment downregulates expression of STAT3 in murine breast cancer **(A)** Analysis of STAT3 expression in 4T1 cells exposed to DE-EDCP or cisplatin (31.25 μM) for 24h using flow cytometry by first gaiting out cell debris and cell clumps in forward/side scatter plot. Data are presented as the mean ± SD (^*^ DE-EDCP vs. untreated p=0.002; DE-EDCP vs. cisplatin p=0.002). Representative histograms of three independent experiments are shown. **(B)** mRNA expression of STAT3, NANOG and SOX2 quantified by RT-PCR in 4T1 cells after DE-EDCP (31.25 μM) 24h treatment. β-actin mRNA was used as an internal control. Data points are represented by the expression ratio and mean±SD fold of control in 4T1 cells. (^*^ STAT3-: DE-EDCP vs. untreated p=0.008; DE-EDCP vs. cisplatin p=0.023; NANOG-: DE-EDCP vs. untreated p=0.019; DE-EDCP vs. cisplatin p=0.029; cisplatin vs. untreated p=0.001; SOX2-: DE-EDCP vs. untreated p=0.021;. cisplatin vs. untreated p=0.021). **(C, D)** At 36^th^ day of the experiment, tumors were harvested from tumor-bearing mice treated with DE-EDCP, cisplatin and vehicle and phospho-STAT3 expression was detected using immunohistochemical method. Representative images and quantitative analysis of the percentage of phospho-STAT3-positive cells are shown. Phospho-STAT3-positive cells were counted in five random fields (magnification at x 400), and data were summarized as the mean percentage of positive cells (four tumors per group). Data are presented as mean ± SE.

DE-EDCP decreased expression of STAT3 (18.0%±3.05) in 4T1 cells in comparison to cisplatin treated (41.23%±12.18) and untreated cells (42.4%±18.62) (Figure 7A). In line with this finding, the marked decrease of STAT3 mRNA level was observed in 4T1 cells treated with DE-EDCP (Figure 7B). Reduction in STAT3 expression after DE-EDCP treatment was accompanied with significantly lower expression of NANOG and SOX2 mRNA, as one of downstream targets of STAT3 signaling pathway (Figure 7B). However, the percentage of Y705 phospho-STAT3-positive cells within breast cancer tissue of DE-EDCP and cisplatin treated mice was decreased compared to control group, although this difference did not reach statistical significance (Figure 7C and 7D).

### DE-EDCP at its effective concentration was well tolerated *in vivo*

Finally, in order to examine the safety of DE-EDCP *in vivo*, the mice's body weight was assessed before and 36^th^ day after tumor cell inoculation. Also, on 36^th^ day after tumor cell inoculation the serum levels of transaminases (AST and ALT), creatinine and urea were measured.

**Figure 8:**
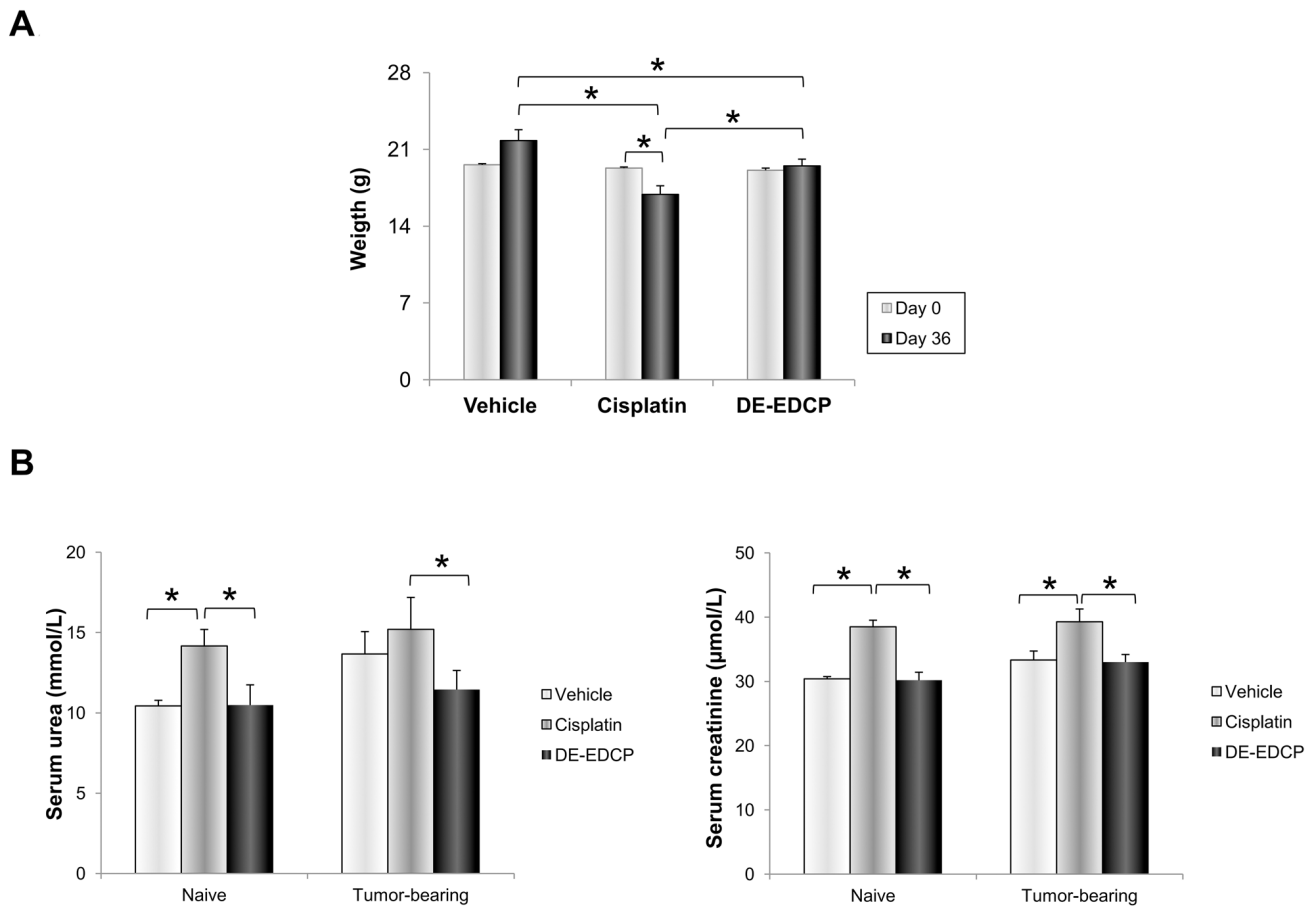
Administration of DE-EDCP is accompanied by fewer side effects compared to cisplatin **(A)** Body weight of tumor-bearing mice treated with DE-EDCP, cisplatin or vehicle before and 36^th^ day of experiment (^*^ DE-EDCP day 36 vs. vehicle day 36 p=0.023; DE-EDCP day 36 vs. cisplatin day 36 p=0.039; cisplatin day 36 vs. vehicle day 36 p=0.002; cisplatin day 0 vs. cisplatin day 36 p=0.03). **(B)** Levels of serum urea and creatinine among cisplatin, DE-EDCP and vehicle treated naive and tumor-bearing mice (n=12-14 animals per group). Data are presented as mean ± SD. (^*^creatinine levels-: naive control group cisplatin vs. vehicle p=0.030; cisplatin vs. DE-EDCP p=0.029; tumor-bearing group cisplatin vs. vehicle p=0.030; cisplatin vs. DE-EDCP p=0.029; ^*^urea levels-: naive control group cisplatin vs. vehicle p=0.005; cisplatin vs. DE-EDCP p=0.005; tumor-bearing group cisplatin vs. DE-EDCP p=0.005).

As shown in Figure 8A, DE-EDCP administration resulted in significant weight loss at the end of treatment. However, DE-EDCP induced reduction of body weight was lower in comparison with cisplatin treatment. Mice treated with cisplatin lost approximately 22% vs. 10% of weight induced by DE-EDCP 36^th^ day after tumor cell inoculation (Figure 8A). Further, serum levels of creatinine and urea of both naive and tumor-bearing mice treated with cisplatin were significantly increased compared to DE-EDCP treated or untreated mice (Figure 8B). It appears that DE-EDCP did not significantly affected systemic levels of creatinine and urea.

There were no significant differences in levels of transaminases (data not shown). Additionally, histological changes were not observed in organs of mice treated with DE-EDCP, including the liver, kidney, lung, intestine, spleen and stomach (data not shown). Taken together, obtained data indicate that DE-EDCP administration is accompanied with fewer side effects when compared to cisplatin.

## DISCUSSION

The major limitations in cancer treatment are drug resistance and severe side effects of conventional chemotherapeutics. Due to limitations related to the treatment of cancer, there is an opening field for the development of novel therapeutic compounds exhibiting higher efficiency as well as lower toxicity. Herein, anticancer activities of newly synthetized compound DE-EDCP were investigated using murine breast cancer model. Testing cytotoxicity of organic ligands and their corresponding platinum complexes revealed that DE-EDCP affected the viability of several cancer cell lines similarly or even efficiently then cisplatin [14–16]. In fact, DE-EDCP exhibited higher cytotoxic capacity compared to cisplatin, especially against human acute myelogenous (KG-1) and promyelocytic leukemia (HL-60) as well as murine melanoma (B16) cell lines [14–16]. These data implicate that DE-EDCP might be considered as a valuable candidate for anticancer therapy. However, to date the effects of DE-EDCP on tumor growth and metastasis are unexplored.

DE-EDCP exhibited significant dose-dependent cytotoxic effect against murine (4T1) and human (MDA-MB-468 and MDA-MB-231) breast cancer cell lines (Figure 1). The most important finding to emerge from present study is that DE-EDCP is capable of favorably influencing the course of malignant disease even after “therapeutic” administration to mice that have already developed tumor. DE-EDCP exhibited marked anticancer activity by reducing breast cancer growth and progression as evaluated by significantly lower tumor volume and weight (Figure 2A) as well as decreased number and size of lung and liver metastases when compared to untreated group (Figure 2B, 2C and 2D). Also, it should be noted that until the 22^th^ day of experiment, DE-EDCP exhibited similar effects on tumor growth as cisplatin (Figure 2A). After that point, cisplatin showed better effects which could be attributed to possible differences in pharmacokinetic factors. It is known that cisplatin, as metal-based anticancer drug, has low platinum excretion rate and is retained in the body, thus achieving its effects in a longer period of time, especially when nephrotoxicity occurs [4]. However, pharmacokinetic properties of DE-EDCP remain unknown. Therefore, it seems quite arbitrary to *assume* that application of DE-EDCP, according to its organic chemical structure, should not conduct to progressive cellular accumulation thus potentially avoiding side-effects. As opposed, cellular accumulation of cisplatin, especially the relatively high degree of accumulation in the renal tissue, might lead to diverse side-effects such as cisplatin-induced nephrotoxicity.

It is well known that dysregulations of apoptosis and cell proliferation are key events in cancer development. Compounds that promote apoptosis and inhibit dysfunctional cell proliferation efficiently prevent the cancer growth and progression. As a conventional chemotherapeutic, cisplatin may trigger the activation of both intrinsic and extrinsic pathway of apoptosis [4]. Therefore, the next aim of the present study was to investigate the possible mechanisms underlying the cytotoxic capacity of DE-EDCP. Initially, it was observed that 4T1 cells exposed to various concentrations of DE-EDCP for 24 hours undergo significant morphological changes indicating that cell death might occurs via apoptosis (Figure 3A). In addition, the expression of important counterparts in apoptotic cell death such as anti-apoptotic Bcl-2, pro-apoptotic Bax or cleaved caspase-3 [19] was observed in both DE-EDCP- and cisplatin-treated 4T1 cells as evaluated by immunofluorescence (Figure 3B). In line with these findings, treatment with DE-EDCP or cisplatin downregulate mRNA level of Bcl-2 expression and upregulate of Bax and caspase-3 mRNA (Figure 3C). Further, DE-EDCP (31.25 and 62.5 μM) significantly increased the percentage of late apoptotic Ann V^+^PI^+^ 4T1 cells in dose-dependent manner following 24 hours treatment (Figure 4B). The highest percentage of late apoptotic cells was observed after DE-EDCP treatment at the concentration of 62.5 μM compared to untreated, but also to cisplatin treated cells. In addition, significantly elevated percentage of TUNEL-positive nuclei in breast cancer tissue following exposure to DE-EDCP or cisplatin (Figure 4C and 4D). In accordance with these findings, it was recently established that DE-EDCP (50 μM) enhanced the superoxide anion production and inner mitochondrial membrane depolarization as evaluated by detection of phosphatidylserine externalization and DNA fragmentation due to apoptosis of HL-60 cells [16]. Pt(II) complex containing DE-EDCP induced HL-60 oxidative stress and apoptosis further evidenced by activation of initiator caspases (caspase-8 and caspase-9) [15]. On the other hand, Pt(IV) complex containing DE-EDCP causes oxidative stress-mediated necrotic death of glioma, melanoma and fibrosarcoma cells *in vitro* [14]. It appears that DE-EDCP and corresponding platinum(II) or platinum(IV) complexes may trigger at least two different types of cancer cell death. However, the authors do not exclude the possibility that these difference could be due to some specific properties of investigated cell lines. Herein, the results obtained from LDH assay strongly implicate that DE-EDCP, as opposed to cisplatin, affects tumor cell membrane integrity as an alternative way for the induction of cell death. Overall, the presented data strongly implicate cytotoxic activity of DE-EDCP *in vitro* and *ex vivo.*

There are evidences that some anticancer agents affect cancer cell proliferation by arresting their cell cycle in G0/G1 phase [20, 21]. Initially, it was noticed that DE-EDCP alters cell cycle status by arresting the 4T1 cells in G0/G1 phase thus preventing cell cycle progression (Figure 5A). Although cyclin E is essential for progression through the G1-phase of the cell cycle [22], DE-EDCP treatment did not affect the percentage of cyclin E-positive 4T1 cells (Figure 5B). Cyclin D3, as initial activator of G1/S phase, is one of the critical players that drives cell proliferation [23, 24]. Overexpression of cyclin D3 has been reported in invasive breast cancer and represents an independent prognostic marker [25]. It has been demonstrated that the beneficial anticancer effect of rapamycin is mainly based on the arresting cells in G1 phase due to destabilization and subsequent down-regulation of cyclin D3 protein in HER2-overexpressing breast cancer cells [26]. Accordingly, down-regulation of cyclin D3 at both the protein and mRNA levels in 4T1 cells was noticed following treatment with DE-EDCP when compared to untreated cells (Figure 5C). Besides cyclins, cyclin-dependent kinases and cyclin dependent kinases inhibitors tightly regulate the cell cycle progression. DE-EDCP caused significant upregulation of CIP/KIP (p21 and p27) and INK (p16) cyclin-dependent kinase inhibitor levels (Figure 5D). p16 specifically binds CDK4 or CDK6 and inhibits cyclin D association in early stage of G1 phase [27]. p21 is a universal cyclin-CDK inhibitor [28], while p27 prevents the activation of cyclin A-Cdk2, cyclin E-CDK2 and cyclin D-CDK4 complexes [27, 29]. The results indicate that DE-EDCP induced G1 cell cycle arrest through downregulating the expression of cyclin D3 and upregulating the expression of p16, p21 and p27.

Ki-67, as widely used marker of cell proliferation, is tightly linked to the cell cycle machinery but its exact role is still unknown. This nuclear protein is expressed in all active phases of cell cycle, but not in quiescent or resting cells in the G0 and early G1 phase [30, 31]. For this reason, Ki-67 could be used as prognostic biomarker and metastatic predictor in many type of human malignancies [32–34]. However, recent study reports that Ki-67 is responsible for cancer resistance to chemotherapeutic agents via maintaining the cancer stem cell niche [17], and suggests that Ki-67 is an attractive therapeutic target in cancer. Our data showed that the percentage of Ki67-positive 4T1 cells exposed to DE-EDCP was markedly decreased compared to untreated and cells exposed to cisplatin (Figure 6A). Decreased expression of Ki-67 in DE-EDCP treated 4T1 cells could be the result of G0/G1 cell cycle arrest and increased expression of CDK4/CDK6 inhibitor, p16, as it was previously shown in normal and cancer cells [35]. Unexpectedly, the highest percentage of Ki-67-positive cells was observed after cisplatin treatment (Figure 6A). Further, immunohistochemistry confirms that DE-EDCP reduced Ki-67 expression in breast cancer *ex vivo*, but there was no difference in the expression of Ki-67 in breast cancer from mice treated with cisplatin or vehicle. We speculate that the higher percentage of Ki-67-positive cells following cisplatin treatment *in vitro* is mainly related to apoptosis-induced compensatory proliferation [36]. This is in line with the results presented above which demonstrated that cisplatin was more effective inducer of 4T1 cell apoptosis at concentration as low as 31.25 μM.

Signal transducer and activator of transcription 3 (STAT3) is constitutively activated in numerous cancers and contributes to different processes involved in tumor progression such as cell proliferation, angiogenesis and metastasis [18, 37]. Therefore, STAT3 could be considered as an important target for cancer treatment. In addition, lower activity of STAT3 lead to G0/G1 cell cycle arrest and apoptosis [38]. The present study revealed that DE-EDCP significantly reduces the percentage of STAT3-positive 4T1 cells in comparison with untreated or cisplatin treated cells (Figure 7A). Also, downregulation of STAT3 mRNA in breast cancer cells was noticed following treatment with DE-EDCP when compared to both untreated and cisplatin exposed cells (Figure 7B). It has been reported that knockdown of STAT3 protein in 4T1 cell line by small interfering RNA (siRNA) reduce expression of c-Myc and completely block expresion of Twist protein, thus inhibiting tumor growth and metastasis [39]. Accordingly, it seems that one of important anticancer effects of DE-EDCP is the reduction of STAT-3 expression in cancer cells.

Recent results reported by Wang H et al [40] suggested that SOX2 and NANOG are the key downstream proteins of STAT3 signaling pathways in cancer. SOX2 and NANOG are transcription factors and biomarkers for cancer stem cells (CSCs) [41, 42]. Also, evidence has revealed that the SOX2 and NANOG are critical factors in conferring certain CSC properties to cancer cells such as self-renewal, metastasis and drug resistance [43, 44]. Aberrant expression of NANOG has been previously found in a variety of tumors, including breast cancer [45]. The upregulation of NANOG expression is linked to tumor progression and relapse [44, 45]. High SOX2 expression was related to adverse breast carcinoma profile, less differentiated subtype and poor disease outcome [46]. Our data showed that DE-EDCP treatment significantly reduces NANOG and SOX2 mRNA level in 4T1 treated cells compared to untreated cells (Figure 7B). Decreased gene expression of stemness transcription factors accompanied with lower expression of Ki-67 in DE-EDCP treated 4T1 cells is in agreement with previous report [17]. In addition, in line with finding that DE-EDCP treatment markedly reduced expression of STAT3 at protein and mRNA level *in vitro*, immunohistochemistry analysis showed a similar trend for decreased percentage of Y705 phospho-STAT3-positive cells within breast cancer tissue of DE-EDCP treated mice compared to control group, although this difference did not reach statistical significance (Figure 7C and 7D). A previous report demonstrated that knockdown of STAT3 protein in cervical carcinoma cell line by siRNA reduced mRNA and protein expressions of NANOG and SOX2 [40]. Accordingly, data obtained from our study indicated that reduction in STAT3 expression after DE-EDCP treatment resulted in significantly lower expression of NANOG and SOX2 mRNA.

The main problems with approved anticancer drugs today are drug-resistance and lack of selectivity responsible for numerous toxic side effects. It is well known that cisplatin treatment is associated with nephrotoxicity, hepatotoxicity and cardiotoxicity, as well as loss in body weight [4]. Results obtained from our study demonstrated that DE-EDCP, in effective concentration of 10 mg/kg, was well tolerated *in vivo*. DE-EDCP induced reduction of body weight was lower in comparison with cisplatin treatment (Figure 8A). We have also observed higher serum levels of both creatinine and urea of cisplatin treated mice in comparison to DE-EDCP treated mice. Although this side effect of cisplatin is well known, the molecular mechanisms underlying cisplatin nephrotoxicity is still under investigation [47–49]. On the contrary, DE-EDCP did not increase serum levels of both creatinine and urea compared to untreated animals (Figure 8B). This lack of nephrotoxicity may be the consequence of DE-EDCP chemical structure as an organic ester. Taken together, obtained data indicate that DE-EDCP administration is accompanied with fewer side effects when compared to cisplatin.

Collectively, DE-EDCP administration exhibits respectable anti-cancer activity with fewer side-effects in comparison with conventional chemotherapeutic. It appears that tumoricidal capacities of DE-EDCP may be achieved by several mechanisms such as triggering cancer cell death via apoptosis or nonapoptotic cell death induced by direct interfering with cancer cell membrane, and inhibition of cell proliferation. The tumoricidal and anti-proliferative capacities of DE-EDCP might be of interest in development of new anticancer agent.

## MATERIALS AND METHODS

### Chemicals

**Figure 9:**
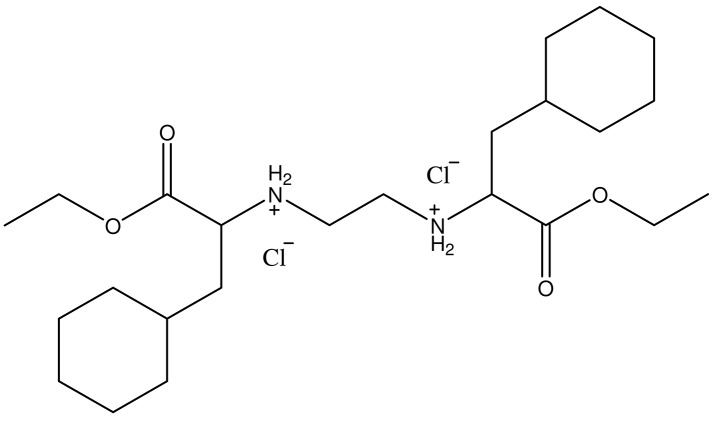
Chemical structure of *O,O’-*diethyl-(*S,S*)-ethylenediamine-*N,N’*-di-2-(3-cyclohexyl)propanoate dihydrochloride

The substance, *O,O’*-diethyl-(*S,S*)-ethylenediamine-*N,N’*-di-2-(3-cyclohexyl)propanoate dihydrochloride (compound marked as DE-EDCP, Figure 9), was synthesized according to previously described procedure [14, 15] and registered as patent in Intellectual Property Office of the Republic of Serbia (Number 2015/11427-P-2013/0270). Cisplatin (cis-diamminedichloroplatinum(II)/cis-[PtCl_2_(NH_3_)_2_]) (Sigma-Aldrich) was used without purification.

### Cell culture

4T1 mouse mammary carcinoma cell line was purchased from American Type Culture Collection (CRL-2539^TM^; ATCC, USA). These cells were routinely grown in suspension in complete DMEM medium in a 5% CO_2_ incubator with standard conditions. In all *in vitro* and *in vivo* experiments only cell suspensions with >95% viable cells were used. Trypan blue was used to determinate the number of viable tumor cells.

### Cytotoxicity assays

#### MTT assay

4T1, MDA-MB-231 and MDA-MB-468 cells were seeded on 96-well plates at a density of 3 × 10^3^ cells/100 μl (per well) in complete DMEM (Invitrogen) growth medium and were allowed to adhere by incubation at 37°C overnight. After 24 hours, culture medium were replaced and each well received 100μl of different compounds, which had been serially diluted two-fold in medium to concentrations ranging from 1000 to 0.49 μM. Cells were incubated under standard conditions (37°C/5% CO_2_) for 24 and 48 hours. Upon incubation medium was removed, MTT (3-(4,5-dimethylthiazol-2-yl)-2,5-diphenyltetrazolium bromide) solution (5mg/ml in PBS, 20 μl) was added to each well and the 96-multiplates were incubated for additional 4 hours. Using microplate multimode detector Zenyth 3100 the optical density of each well was determined at 595 nm. The percentage of cell viability was determined by comparison with untreated controls according to formula: % of viable cells = (E-B)/(S-B) x 100 where B is for background of medium alone, S is for total viability/spontaneous death of untreated target cells, and E is for experimental well [50]. Experiments, performed in triplicates, were repeated three times.

#### LDH assay

Cytotoxicity of test compound was examined by *In Vitro* Toxicology Assay Kit (Lactic Dehydrogenase based) (Sigma-Aldrich, St. Louis, MO). Cells were prepared and treated with test compound in the same way as for MTT assay. A high control, leading to 100% cytotoxicity by lysing the cells completely, was included in the assay. Cells exposed to medium only were used as a low control. After 24 hours of treatment, supernatant (50 μl) was transferred to new multiplate and incubated with previously prepared substrate solution (100 μl). Plates were protected from light and incubated at room temperature for 30 minutes. The reaction was stopped by stop solution and data were acquired by spectrophotometry at 490 nm. Experiments were repeated three times. The percentage of dead cells was calculated using the formula [51]:

% dead cells = (exp. value-low control)/(high control-low control) x 100

### Annexin V propidium iodide double staining assay

Annexin V is cellular protein with ability to bind to phosphatidylserine when it is on the external portion of the plasma membrane. Propidium iodide (PI), as small fluorescent molecule, binds to DNA but cannot passively traverse into cells if plasma membrane remains intact [52]. After 24 hours of treatment with test compound at concentrations 31.25 and 62.5 μM, 4T1 cells were stained with Annexin V-FITC, followed addition of PI (BD Pharmingen, San Diego, California, USA) according to the manufacturer's instructions. The percentages of dead cells were determined by FACS Calibur flow cytometer (BD Biosciences, San Jose, USA) and the data were analyzed using FlowJo (Tree Star). It is considered that Annexin V^-^ PI^-^ are viable cells, Annexin V^+^PI^-^ are cells in early stage of apoptosis, Annexin V^+^ PI^+^ are cells in late stage of apoptosis, while Annexin V^-^ PI^+^ are necrotic cells.

### Cell cycle analysis

4T1 cells were allowed to grow until 70–80% confluent in culture plates and were exposed to DE-EDCP or cisplatin (31.25 and 62.5 μM) for 12 hours and cell cycle analysis was performed with Vybrant® DyeCycle™ Ruby stain (Thermo Fisher Scientific, Inc. USA) according to manufacturer's instructions. After treatment, 4T1 cells were stained with Vybrant DyeCycle Ruby and analyzed by FACS Calibur flow Cytometer (BD Biosciences, San Jose, USA). The cell cycle distribution was analyzed using FlowJo software [53].

### Flow cytometric analysis

4T1 cells, grown in culture plates, were treated with either DE-EDCP or cisplatin (31.25 μM) for 24 hours. Next, the treated cells were fixed and permeabilized with permeabilization buffer (BD Bioscience) and incubated with antibodies specific for STAT3 (IC1799G, Novus Biologicals, San Diego, USA), Ki-67 (11-5698-82, eBioscience, San Diego, USA), cyclin D3 (ab28283, Abcam Cambridge, United Kingdom), cyclin E (MA5-14336, Thermo fisher scientifics, USA), p16 (ab211542, Abcam Cambridge, United Kingdom), p21 (ab188224, Abcam Cambridge, United Kingdom) and p27 (ab215434, Abcam Cambridge, United Kingdom). For staining cyclin D3, cyclin E, p16, p21 and p27 cells were additionally incubated with secondary goat anti-mouse IgG FITC (ab6785 Abcam Cambridge, United Kingdom) or donkey anti-rabbit IgG (ab150073 Abcam Cambridge, United Kingdom). Flow cytometry was conducted on FACSCalibur flow cytometer (BD Biosciences, San Jose, USA) and the data were analyzed using FlowJo (Tree Star).

### Immunofluorescencent staining

The expression of Bcl-2, Bax and cleaved caspase-3 proteins were investigated by immunofluorescence method as previously described [54]. In brief, the 4T1 cells were seeded in a 6-well plate and exposed to the DE-EDCP and cisplatin at concentration of 31.25 μM for 24 hours. After washing the cells twice with PBS, they were fixed in 4% paraformaldehyde at 25°C for 20 min. The cells were stained with rabbit polyclonal antibody specific for Bcl-2 (sc-783, Santa Cruz Biotech. Inc CA, USA), Bax (sc-493, Santa Cruz Biotech. Inc CA, USA) and active/cleaved caspase-3 (NB100-56113, Novus Biologicals, UK). After incubation, the cells were washed and treated with appropriate secondary antibody, goat anti-rabbit IgG FITC (Ab6717-1, Abcam, Cambridge, United Kingdom). The sections were mounted with ProLong Gold antifade reagent with DAPI (Invitrogen) and analyzed at x 200 magnification using fluorescent microscope (Olympus BX 51).

### Real-time PCR analysis

4T1 cells were treated with DE-EDCP and cisplatin (31.25 μM) for 24 hours. Total RNA from 4T1 cell was isolated with TRIzol (Invitrogen, Carlsbad, CA). First-strand cDNA was synthesized by High Capacity cDNA Reverse Transcription Kit (Applied Biosystems, Foster City, California, USA). qRT-PCR was performed using Power SYBR MasterMix (Applied Biosystems) and mRNA specific primers for Bax (forward 5´-ACACCTGAGCTGACCTTG-3´ and reverse 5´-AGCCCATGATGGTTCTGATC-3´), Bcl-2 (forward 5´-GTGGTGGAGGAACTCTTCAG -3´ and reverse 5´-GTTCCACAAAGGCATCCCAG-3´), Caspase-3 (forward 5´-AAATTCAAGGGACGGGTCAT-3´ and reverse 5´-ATTGACACAATACACGGGATCTGT-3´), STAT3 (forward 5´-GGCATTCGGGAAGTATTGTCG-3´ and reverse 5´- GGTAGGCGCCTCAGTCGTATC-3´), Cyclin D3 (forward 5´- CCGTGATTGCGCACGACTTC-3´ and reverse 5´-TCTGTGGGAGTGCTGGTCTG-3´), NANOG (forward 5´-AAGCAGAAGATGCGGACTGT-3´ and reverse 5´-GTGCTGAGCCCTTCTGAATC-3´), SOX2 (forward 5´-AAAGGGTTCTTGCTGGGTTT-3´ and reverse 5´-AGACCACGAAAACGGTCTTG-3´) and β-actin (forward 5´-AGCTGCGTTTTACACCCTTT-3´ and reverse 5´-AAGCCATGCCAATGTTGTCT -3´) as a housekeeping gene. qRT-PCR reactions were initiated with a 10 minute incubation time at 95°C followed by 40 cycles of 95°C for 15 seconds and 60°C for 60 seconds in a Mastercycler ep realplex (Eppendorf, Hamburg, Germany). For each sample (four per group) relative amount of mRNA was normalized to the β-actin content. Fold expression changes relative to 4T1 cells were calculated with the ΔΔCT method [55]. Data points are represented by the expression ratio and mean±SD fold of control in 4T1 cells.

### Experimental animals

All experiments were approved by and conducted in accordance with the Guidelines of the Animal Ethics Committee of the Faculty of Medical Sciences of the University of Kragujevac, Serbia. Female (8–10 weeks old) BALB/c mice were used. The mice were housed in a temperature-controlled environment with a 12-hour light-dark cycle, fed *ad libitum* and observed daily. Also, experimental animals were equalized in weight and randomized in experimental or control groups.

### Animal model and drug treatment

#### Orthotopic breast cancer model

BALB/c mice were inoculated with 3 × 10^4^ 4T1 cells orthotopically into the fourth mammary fat pad. Five days after 4T1 cells were applied, the mice were injected with a chemotherapy drug by intraperitoneal injection. Female BALB/c mice received either DE-EDCP (10 mg/kg body weight/ for 5 consecutive days followed by 2 days break and treated again for 5 days), cisplatin (3mg /kg/dose; three times per week; nine doses in total) or phosphate-buffered saline. Mice were sacrificed on 36^th^ day of the experiment.

### Estimation of breast cancer growth

The size of primary 4T1 mammary tumors was assessed morphometrically using electronic calipers in two dimensions. Using a below formula the tumor volumes (mm^3^) were calculated [56]: tumor volume (mm^3^)=L (major axis of the tumor) x W(minor axis)^2^/2.

### Histopathological analysis of metastatic lung and liver

Hematoxylin-eosin (H&E) staining was performed using 4 μm paraffin-embedded tumor bearing lung or liver sections. In order to avoid missing micrometastases, stained sections from at least three different levels were examined for the presence of lung or liver metastases. Metastases were verified at magnification x 100 and x 400 and photomicrographed with a digital camera mounted on light microscope (Olympus BX51). In the histological section metastatic area per tissue was calculated using ImageJ software [57].

### *In situ* TUNEL staining

The TUNEL (terminal deoxynucleotidyl transferase mediated dUTP nickend labeling) reaction was performed to assess apoptotic cells within breast cancer tissues sections. Formalin-fixed, paraffin-embedded tissue sections were stained with Apo-tag TUNEL assay kit (Millipore, Temecula, CA, USA) following the protocol of manufacturer. DAB (3,3’-diaminobenzidine) as peroxidase substrate, was used to yield the characteristic brown color for nuclei. Following rinsing, slides were counterstained with hematoxylin solution and photomicrographed with a digital camera mounted on light microscope. The negative control was performed by omitting TdT reaction step. The TUNEL-positive nuclei (brown) were quantified under × 400 magnification in five randomly fields representing at least 1000 neoplastic nuclei and the data were summarized as the mean percentage of positive cells (four tumors per group) [58].

### Immunohistochemical detection of Ki-67 and phospho-STAT3 (pSTAT3) in breast cancer tissues

Briefly, paraffin-embedded breast cancer tissue sections were deparaffinized, rehydrated and subjected to heat-induced antigen retrieval in citrate buffer (pH 6.0). Next, sections were incubated with rabbit anti-mouse Ki-67 antibody (ab66155, Abcam, Cambridge, United Kingdom) or STAT3 (phospho Y705) antibody (ab76315, Abcam, Cambridge, United Kingdom) followed by visualization using the rabbit-specific conjugate (Expose Rb-Specific HRP/AEC Detection IHC Kit; Abcam Cambridge, United Kingdom). The slides were photomicrographed with a digital camera mounted on light microscope. The negative control slides were obtained by omitting the primary antibody.

The Ki-67-positive, as well as pSTAT3-positive, cells were determined by counting at least 1000 neoplastic nuclei per slide in five randomly selected fields (at magnification x 400). The data were summarized as the mean percentage of positive cells (four tumors per group) [59].

### Toxicity assessment

To study the potential side effects in the DE-EDCP treated mice, they were remarked for weight loss. After sacrificed, tissue sections of various organs (liver, kidney, lung, intestine, spleen and stomach) stained with H&E and were also examined. The levels of serum urea, creatinine and transaminase were determined to assess the renal and hepatic function. After blood collection, serum levels of these toxicity markers were measured immediately using assay kits and blood chemistry analyzer, as described [60].

### Statistical analysis

The data were analyzed using software package IBM SPSS Statistics version 20. First the normality of data distribution was tested by Kolmogorov-Smirnov or Shapirov-Will test. The two-tailed Student's t test or nonparametric Mann–Whitney Rank Sum test were used. All data in this study were expressed as the mean ± standard deviation (SD) or standard error (SE). Values of p < 0.05 were considered as statistically significant.
